# Supplementary material for: Systematic identification of protein combinations mediating chromatin looping
Source: Nat Commun. 2016 Jul 27;7:12249. doi: 10.1038/ncomms12249 (PMC4974460; doi:10.1038/ncomms12249)
Supplement: Supplementary Information — Supplementary Figures 1-2 and Supplementary Tables 1-2 [file ncomms12249-s1.pdf]

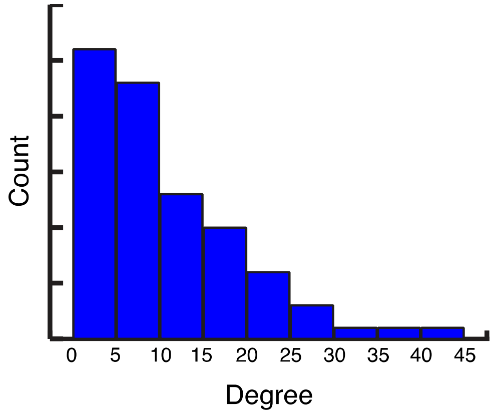

Supplementary Figure 1: Degree distribution of DBP cooperation network in GM12878.

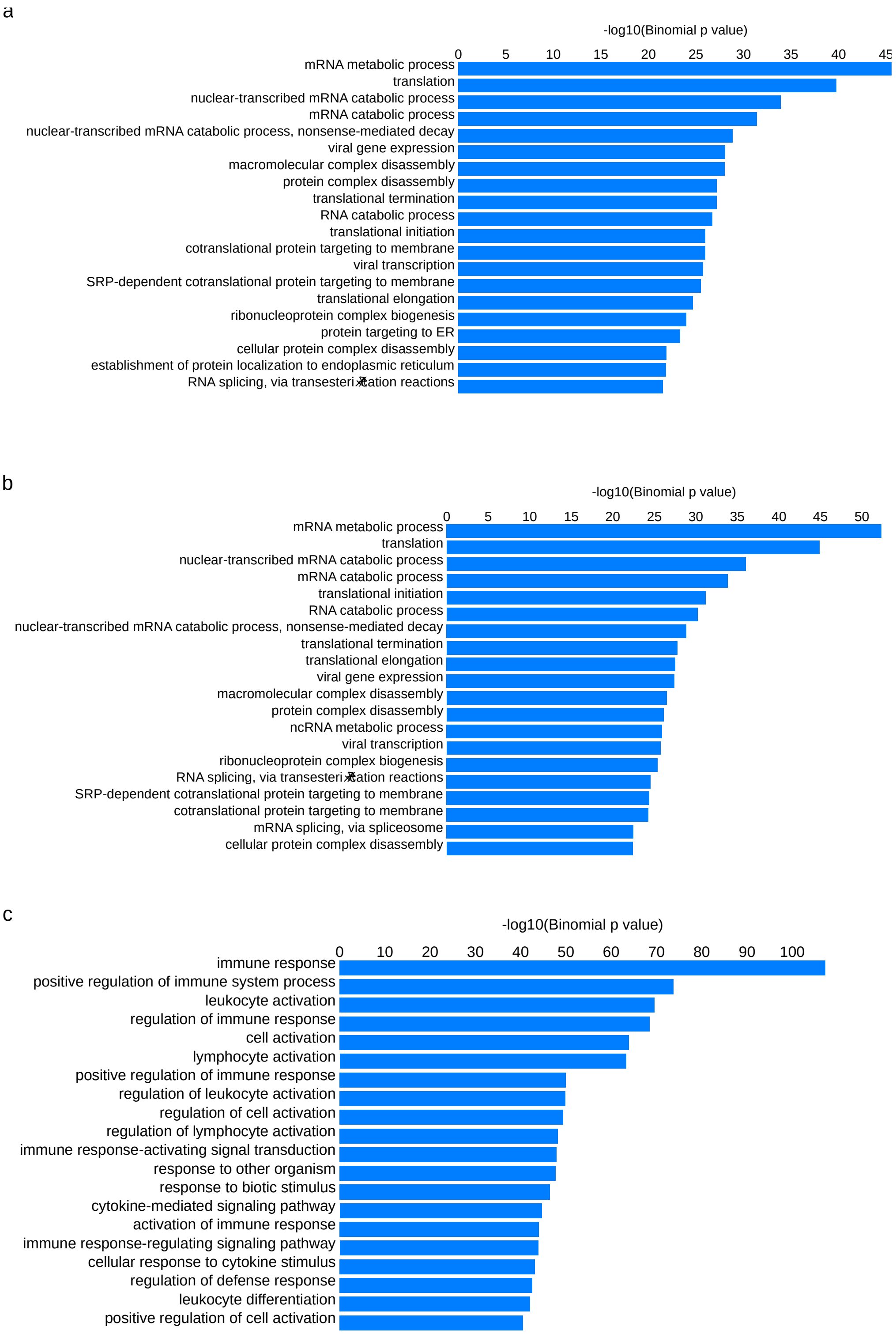

Supplementary Figure 2: Functional analysis of communities in the GM12878 DBP cooperation network. (a) Enriched GO terms in green community. (b) Enriched GO terms in cyan community. (c) Enriched GO terms in red community.

Supplementary Table 1: Speed comparison of GGM and ARACNE on data sets with different sizes.

Algorithm	Number of nodes	Number of samples	Average running time and standard deviation (second)
ARACNE	50	100	0.44(0.001)
		200	1.6(0.001)
		400	6.3(0.003)
	100	100	1.7(0.002)
		200	6.5(0.005)
		400	25(0.03)
	200	100	6.8(0.004)
		200	26(0.005)
		400	102(0.23)
GGM	50	100	0.03(0.002)
		200	0.07(0.001)
		400	0.14(0.001)
	100	100	0.09(0.002)
		200	0.17(0.001)
		400	0.33(0.002)
	200	100	0.34(0.008)
		200	0.49(0.002)
		400	0.89(0.002)

Supplementary Table 2: Fours communities found in the GM12878 DBP cooperation network.

Red community	Cyan community	Green community	Yellow community
ATF2	BCLAF1	EBF1	CTCF
BATF	BHLHE40	EGR1	RAD21
BCL11A	BRCA1	ELF1	SMC3
BCL3	CHD1	EP300	YY1
CEBPB	CHD2	GABPA	ZNF143
FOXM1	CREB1	H2AFZ	
IRF4	CUX1	H3K27ac	
JUND	E2F4	H3K4me1	
MEF2A	ELK1	H3K4me2	
MEF2C	ESRRA	H3K4me3	
MTA3	ETS1	H3K79me2	
NFATC1	MAFK	H3K9ac	
NFIC	MAX	IKZF1	
PML	MAZ	IRF3	
POLR2AphosphoS2	MXI1	MYC	
RELA	NRF1	NFYB	
RUNX3	RCOR1	PAX5	
RXRA	RFX5	PBX3	
SP1	SIN3A	POLR2A	
STAT5A	SREBF1	POLR2AphosphoS5	
	SREBF2	POU2F2	
	STAT1	REST	
	STAT3	SIX5	
	TBL1XR1	SPI1	
	TBP	SRF	
	USF2	TAF1	
		TCF12	
		TCF3	
		USF1	
		WRNIP1	
		ZBTB33	
		ZEB1	
		ZNF384	
